# Ubiquitination and deubiquitination: Implications for the pathogenesis and treatment of osteoarthritis

**DOI:** 10.1016/j.jot.2024.09.011

**Published:** 2024-10-11

**Authors:** Shibo Su, Ruijiao Tian, Yang Jiao, Shudan Zheng, Siqiang Liang, Tianyi Liu, Ziheng Tian, Xiuhong Cao, Yanlong Xing, Chuqing Ma, Panli Ni, Fabiao Yu, Tongmeng Jiang, Juan Wang

**Affiliations:** a Key Laboratory of Tropical Translational Medicine of Ministry of Education & Key Laboratory of Brain Science Research and Transformation in Tropical Environment of Hainan Province, Hainan Provincial Stem Cell Research Institute, School of Basic Medicine and Life Sciences, Hainan Medical University, Haikou, 571199, China; b Department of Biomedical Engineering, Faculty of Engineering, The Hong Kong Polytechnic University, Hong Kong, China; c Plastic Surgery, Second Affiliated Hospital of Hainan Medical University, Haikou, 570100, China; d Zhongke Comprehensive Medical Transformation Center Research Institute (Hainan) Co., Ltd, Haikou, 571199, China; e Department of Pharmacology, Zibo Hospital of Traditional Chinese Medicine, Zibo, 255300, China; f School of Clinical Medicine, Jining Medical University, Jining, 272002, China; g Engineering Research Center for Hainan Bio-Smart Materials and Bio-Medical Devices, Key Laboratory of Hainan Functional Materials and Molecular Imaging, College of Emergency and Trauma, Hainan Academy of Medical Sciences, Hainan Medical University, Haikou, 571199, China; h Key Laboratory of Emergency and Trauma of Ministry of Education, Key Laboratory of Haikou Trauma, Key Laboratory of Hainan Trauma and Disaster Rescue, The First Affiliated Hospital, Hainan Medical University, Haikou, 571199, China; i The Second Clinical College, Hainan Medical University, Haikou, 571199, China

**Keywords:** Chondrocyte, Fibroblast-like synoviocyte (FLS), Macrophage, Osteoarthritis, Osteoblast, Ubiquitination

## Abstract

Osteoarthritis (OA) is a degenerative disease that affects multiple cells and associated extracellular matrix (ECM). Chondrocytes and chondroextracellular matrix together constitute articular cartilage tissue. Any factors that affect the activity of chondrocytes and destroy the metabolic balance of the chondrocyte ECM will lead to the inability of articular cartilage to perform normal functions. The articular subchondral bone and articular cartilage must be coordinated to resist enough friction and mechanical stress, so the articular subchondral bone lesion will aggravate the articular cartilage defect and vice versa. Synoviocytes, including fibroblast-like synoviocytes (FLSs) and synovial macrophages at the joint, are also important factors that cause low-grade chronic progressive inflammation of OA. Regulation of phenotype transformation of synovial macrophages has become another possible target for the clinical treatment of OA. Ubiquitination and deubiquitination are the main post-translational protein modification pathways in the human body, which are widely involved in multiple signaling pathways and physiological processes. Naturally, they also play a very important regulatory role in the occurrence and development of OA. These effects are summarized in this review, including (A) regulating the aging and apoptosis of chondrocytes, FLSs and osteoblasts; (B) regulation of ECM degradation; (C) regulation of macrophage phenotypic transformation; (D) modulation of skeletal muscle and adipose tissues. Ubiquitination targeting drugs for OA treatment are also listed. Depending on the high efficiency of ubiquitination and deubiquitination, understanding OA-related ubiquitination pathways can help design more efficient drugs to treat OA and provide more potential targets for clinical treatment.

The Translational Potential of This Article.

In this paper, the ubiquitination-related pathways in osteoarthritis (OA), including aging, apoptosis and autophagy in chondrocytes, osteoblasts, FLSs and macrophages were investigated. In particular, several ubiquitination-related targets are expected to be effective approaches for OA clinical treatment. In addition, in the process of OA occurrence and development, the complex relationship between the local joint area and other tissues including skeletal muscle and adipose tissue is also discussed. These myokines and adipokines from musculoskeletal tissues are all expected to become efficient targets for OA treatment apart from the joint itself. In addition, those myokines secreted by cardiovascular tissues would show potential therapeutic effects as well. What if altering the contents for these ubiquitination-related targets in the serum through exercise will provide a new idea for OA therapy or prevent OA from deteriorating continuously?

## Graphical abstract

Ubiquitination and deubiquitination participate in the homeostasis between healthy joints and osteoarthritic joints through regulating senescence, apoptosis and autophagy of chondrocytes, fibroblast-like synoviocytes, osteoblasts, macrophages, skeletal muscle and adipose tissue. Digoxin, 6-Gingerol, Resveratrol, Spermidine, Alpinetin and Melatonin are potential therapeutic drugs.

**Figure:**
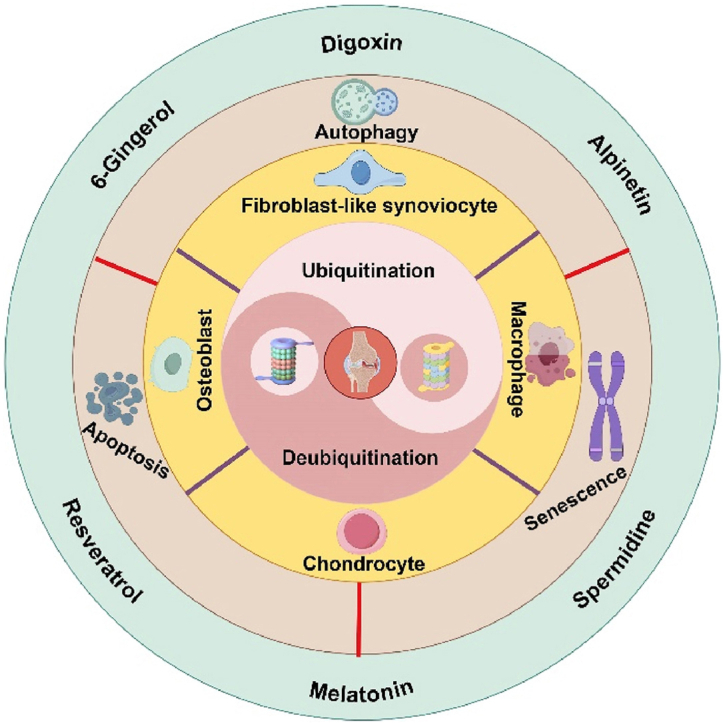

## 1 Introduction

Osteoarthritis (OA) is a chronic, degenerative joint disease [1]. The clinical symptoms are severe joint pain, joint stiffness and deformation, and difficulty bearing weight [2]. Articular cartilage is the main stress recipient during mechanical movement, responsible for counteracting frequent friction and shear stress [3]. In this process, the extracellular matrix (ECM) of chondrocytes is inevitably consumed, but the low turnover of chondrocytes makes it impossible to repair them quickly [4,5]. As a result, excessive ECM defects of chondrocytes occur, the synovial membrane covering the surface of the cartilage is damaged, inflammation occurs, collagen fibers are denaturalized and lose elasticity, and finally, the subchondral bone becomes the main bearer of mechanical stress [6]. In short, excessive destruction and too little repair lead to excessive aging and damage of important joint functional cells such as chondrocytes, osteoblasts, fibroblast-like synoviocytes (FLSs) and synovial macrophages, which are considered to be part of the main reasons for the occurrence and development of osteoarthritis [7]. Because OA involves many factors such as heredity, obesity, gender, occupation, and age differences in adapting to modern environmental conditions, the exact pathogenesis of OA is still unclear [8,9]. However, as for the source of low-grade and progressive inflammation, the existing view is that it is caused by excessive synovial macrophages in the joint cavity and continuous inflammatory polarization [10]. At present, the treatment of OA is mainly through inhibiting pain and reducing inflammation, but it cannot repair cartilage defects and restore normal joint function [11]. Therefore, delaying the aging of chondrocytes, osteoblasts and FLSs, as well as reducing the inflammatory polarization of synovial macrophages will help cure OA fundamentally, which is also of great significance for the development of new therapeutic strategies and effective drugs [12].

Cells can perform normal functions in a timely manner, depending on the accurate expression of genes on the one hand and whether proteins can function normally on the other hand. Under normal circumstances, the polypeptide chain translated by the genetic code in the cell is not functional and needs to be modified by folding, cutting, or some additional chemical changes to achieve normal function, which is called post-translational modification (PTM) [13]. PTM plays a crucial role in regulating biological processes such as cell signaling pathway, cell metabolism, differentiation, metastasis, cell cycle and proliferation [14]. Common PTMs include methylation, ubiquitination, deubiquitination, acetylation, glycosylation, phosphorylation, and SUMOylation [15]. Phosphorylation and ubiquitination are the two most important types, which play an extremely important role in the regulation of cell signaling pathway, cell metabolism, cell cycle and proliferation [16].

**Fig. 1:**
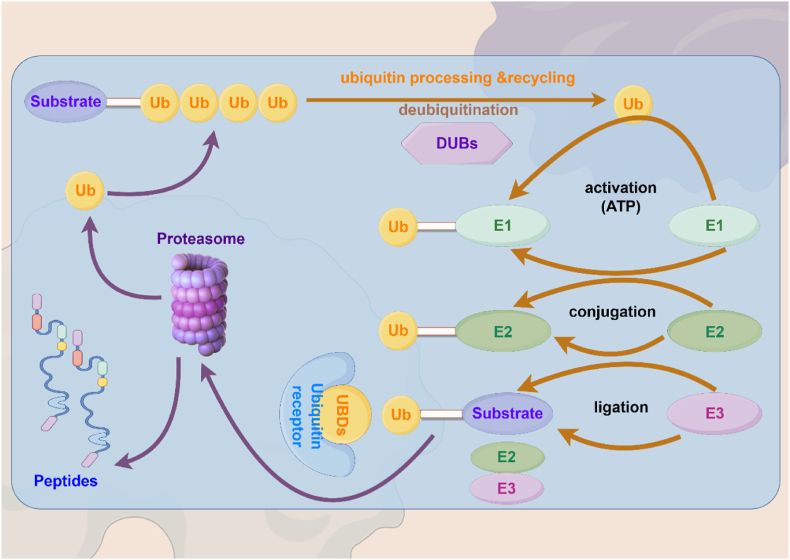
Ubiquitin and deubiquitination reactions. The intricate ballet of ubiquitin reactions unfolds in three meticulously choreographed acts: the activation stage catalyzed by the E1 ubiquitin-activating enzyme, the binding stage facilitated by the E2 ubiquitin-conjugating enzyme, and the immobilization stage executed by the E3 ubiquitin ligase. In the first act, the E1 enzyme, fueled by ATP, awakens ubiquitin molecules from their dormant state, passing the baton to the E2 enzyme in the second act. The grand finale sees the E3 ligase skillfully affixing these activated ubiquitin molecules to substrate proteins, marking them for the proteasome's discerning gaze. Here, the proteins are elegantly cleaved, and the ubiquitin tags are gracefully returned to the pool of free molecules, ready for another performance. Yet, the narrative of ubiquitination is not a one-way street; it is punctuated by the counter-narrative of deubiquitination. This reverse process, akin to an unscripted encore, involves the enzymatic cleavage of ubiquitin from its protein partners, reclaiming the molecules for future roles in the cellular drama. Together, these reactions weave a complex tapestry of protein regulation, ensuring the harmonious symphony of cellular life. By Figdraw.

Ubiquitination is a post-translational modification pathway that involves E1, E2 and E3 enzymes in order to achieve reversible and dynamic regulation of protein levels. During ubiquitination, a specific E1 ubiquitin-activating enzyme first activates Ubiquitin molecules (Ub), and then the activated Ub migrates to the cysteine residue of the E2 ubiquitin-binding enzyme, forming a complex that binds to the E3 ubiquitin ligase, which recognizes and binds to a specific substrate. Ub is also migrated to lysine residues of the substrate [17]. E3 ubiquitin ligase is responsible for recognizing and interacting with specific substrates and thus determines the specificity of the ubiquitination reaction [18]. Ubiquitination-labeled proteins can be mechanically degraded by the proteasome, which is one of the main ways of clearing functional proteins in the cell [19]. The opposite of ubiquitination is deubiquitination. Deubiquitination is performed by deubiquitinases (DUBs). Currently, more than 100 kinds of DUBs have been identified in humans, which can be classified into cysteine protease DUBs and metalloproteinase DUBs according to their catalytic activity [20]. Deubiquitination, the countervailing process to ubiquitination, is of paramount importance, equaling its counterpart in the preservation of protein stability, the maintenance of intracellular free ubiquitin concentrations, the modulation of signal transduction pathways, and the regulation of the cell life cycle [21]. It is clear that for cells to survive and function properly, they must maintain a dynamic balance between ubiquitination and deubiquitination (Fig. 1).

**Fig. 2:**
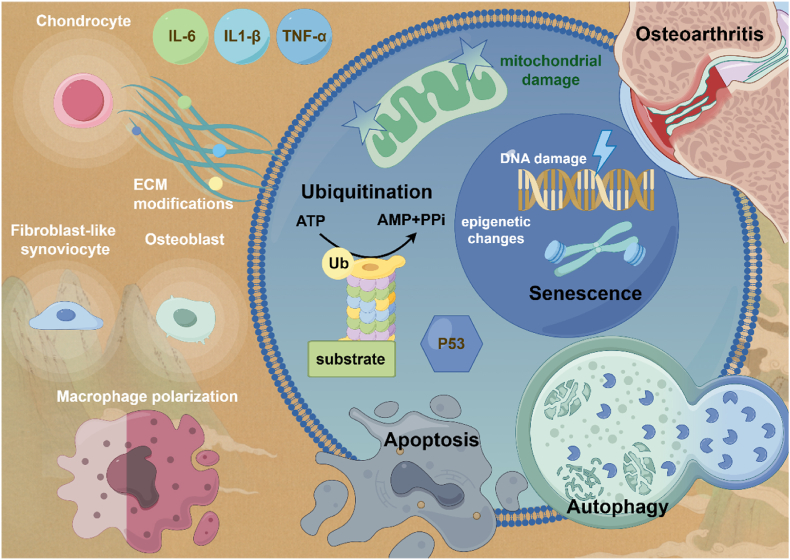
Ubiquitination affects the senescence, apoptosis, and autophagy of chondrocytes, osteoblasts and fibroblast-like synoviocytes (FLSs), as well as modulates the polarization of macrophages in the joints, thus interfering in the occurrence and development of osteoarthritis. By Figdraw.

Recently, ubiquitination and deubiquitination have been found to play an important role in the regulation of a variety of diseases, including osteoarthritis. In this paper, we will explore how ubiquitin can affect the aging and apoptosis of chondrocytes, osteoblasts and FLSs, as well as influence the polarization of synovial macrophages, thus interfering in the occurrence and development of osteoarthritis (Fig. 2). At the same time, the related effects of deubiquitination will be discussed, and the relevant targets of ubiquitination or deubiquitination can be used to move to clinical treatment.

## 2 Ubiquitination regulates chondrocyte senescence, apoptosis and autophagy

Previous studies have shown that chondrocytes/cartilage undergo specific changes of related molecules in the process of degeneration, apoptosis and oxidative stress during OA [22,23]. The accumulation of ROS in OA models indicates that ROS plays an important role in the occurrence and development of OA, while scavenging ROS protects cartilage [24,25]. Mitochondrial metabolism disorder in chondrocytes is the main source of ROS [26]. With the increase of ROS level, on the one hand, negative feedback will continue to damage mitochondria, reduce mitochondrial membrane potential, damage mitochondrial DNA, and then cause mitochondrial function abnormalities and promote apoptosis of chondrocytes [26,27]. On the other hand, low-grade inflammation of chondrocytes and FLSs will be triggered, resulting in up-regulated expression of chondrocyte ECM degrading enzyme and down-regulated expression of chondrocyte specific markers type II collagen and Acan genes, thereby leading to chondrocyte ECM defects, affecting the interaction between matrix and cells and resulting in abnormal joint function [28,29]. Therefore, regulation of mitochondrial metabolic balance and timely removal of damaged and aging mitochondria are potential targets for alleviating oxidative stress and improving OA. PRKN (parkin RBR E3 ubiquitin protein ligase) is an E3 ubiquitin ligase, which is composed of 465 amino acids and multi-domains [30]. If the gene encoding PRKN is mutated, it can lead to Parkinson's disease or autosomal recessive infant Parkinson's disease [30,31]. PINK1, as a serine/threonine kinase containing mitochondrial targeting signals at the N-terminal, interacts with PRKN to form a mitochondrial autophagy mechanism dependent on PRKN ubiquitination [32]. Under normal circumstances, PINK1 needs to be translocated into the inner mitochondrial membrane and undergo multiple cuts before it can function normally. However, when the mitochondria are damaged, the abnormal mitochondrial membrane potential results in the inhibition of the normal translocation and cutting process of PINK1 [33]. A large amount of PINK1 accumulates in the mitochondrial outer membrane and combines with the mitochondrial outer membrane translocation enzyme to form a complex, which will collect PRKN from the cytoplasm to the damaged mitochondria and activate its E3 ubiquitin ligase activity. Multiubiquitination chains are formed on the outer membrane surface of damaged mitochondria, resulting in mitochondrial autophagy [34,35]. The PRKN-PINK1 pathway plays an extremely important role in clearing damage and excess mitochondria while also helping to maintain cell homeostasis, reduce ROS accumulation, and reduce oxidative stress [32]. Although there are no clear results to prove whether the PRKN-PINK1 pathway is helpful in the treatment of OA, PRKN-mediated mitochondrial autophagy is still a possible therapeutic target.

P53 is the most widely studied tumor suppressor protein at present, maintaining the dynamic balance between its synthesis and decomposition is the key to ensuring its normal tumor suppressor function [36]. Cao et al. found that the treatment of OA chondrocytes with the P53 agonist C16-Ceramide resulted in hypertrophy of chondrocytes, reduced synthesis of related polysaccharides and upregulation of SA-β-Gal expression, indicating that P53 was involved in the process of chondrocyte senescence and even apoptosis [37]. The mechanisms by which p53 maintains homeostasis remain elusive, given the complexity of its involvement in regulating multiple PTM pathways. However, Liu Jiang et al. 's recent study found that P53 protein could be covalently modified by UFM1(Ubiquitin-Fold modifier 1), so that P53 protein could avoid ubiquitin labeling and subsequent proteasomal degradation, thus maintaining its stability [38]. UFM1 mediates a similar ubiquitination reaction by covalently adding ubiquitin folding modifiers to specific substrates (intracellular proteins) to trigger the degradation of related proteins. Ufmylation plays a pivotal role in various endoplasmic reticulum (ER)-associated biological processes, including ER-associated protein degradation (ERAD), ribosome-associated protein quality control at the ER, and ER-phagy [39]. It turns out that UFM1 does maintain the stability of P53 by competing with ubiquitination [38]. Intracellular knockdown of UFL1(the E3-like ligase UFM1-specific ligase 1) can significantly reduce P53 protein levels, but does not involve changes in P53 mRNA levels. If cells are treated with proteasome inhibitor MG132, it can prevent the degradation of P53 protein caused by UFL1 knockdown. This suggests that UFM1-mediated ubiquitination of P53 protein antagonizes its ubiquitination process, possibly because UFM1 ubiquititizes four lysine residues on P53 protein that should be ubiquitination sites [39]. Clarifying the related ubiquitination pathway of P53 protein plays a crucial role in inhibiting chondrocyte senescence, and related drugs may be developed to treat OA in the future.

JNK and ERK1/2 have been confirmed to be up-regulated in chondrocytes induced by static load. Li et al. found that injecting lentivirus LINC00313 overexpression vector into the OA mouse model can significantly inhibit the activation of the JNK pathway, and this inhibition effect is apparent as enhanced chondrocyte vitality and reduced apoptosis of chondrocytes. OA inflammation decreased and cartilage extracellular matrix degradation decreased, which could be reversed by JNK activators [40]. The way in which ERK1/2 promotes apoptosis is mainly dependent on caspase-3, cleaved caspase-3 and Caspase-3-7 proteins [41]. Tankyrase (TNKS) is necessary for JNK signaling activation and has a significant regulatory effect on the activity of JNK signaling molecules. Li et al. 's studies have shown that TNKS can mediate K63 polyubiquitination at K151R and K158R sites on JNK. Although K63 polyubiquitination does not aim to degrade core proteins, it can also restrict the activity of JNK, thereby regulating JNK signaling pathway [42]. MEKK1 is the upstream molecule that activates JNK1/2 signaling pathway, but its N-terminal has a PHD(plant homeodomain) domain, and this PHD domain has a ring finger domain, which can act as E3 ligase and promote the polyubiquitylation of ERK1/2 [43].

The results showed that the degree of apoptosis of chondrocytes showed a strong positive correlation with the expression level of fibronectin. Fibronectin is mainly located in the extracellular matrix surrounding chondrocytes and mediates the interaction between cells and ECM through multiple intracellular and extracellular domains [44]. In addition, the expression of fipronectin (FN1) gene was significantly up-regulated in OA chondrocytes, in which the transcript of FD1-208 generated by the expression of FN1 gene stood out among many different transcripts, and artificially up-regulated FD1-208 could simulate the characteristics of OA cartilage [45]. The effect of fibronectin on chondrocyte senescence is complex, and the dynamic change of its transcript ratio regulates the binding of chondrocytes to different ECM macromolecules. In articular cartilage, chondrocytes account for 5 %, and the remaining 95 % are chondrocyte ECM [46]. Such a large difference in content proves the importance of the interaction between chondrocytes and ECM, and the change of this connection is sufficient to affect the aging and apoptosis of chondrocytes [47]. In articular cartilage, the two enzymes that mainly affect the chondrocyte extracellular matrix are matrix metalloproteinases (MMPs) with collagenase activity and platelet reactive proteolytic integrin metallopeptidase 5 (ADAMTS5) with glycosidase activity [48]. Among MMPs, MMP13 is the most widely studied and has the greatest influence on the ECM of cartilage, and it can play a normal role only after MMP14 on the surface of the cartilage cell membrane is cut [49]. F-box protein is a specific substrate recognition component of Skp1-Cullin1-Fbox (SCF)E3 ubiquitin ligase and is involved in multiple life activities including cell growth, apoptosis, differentiation, autophagy, cell cycle and cell migration [50,51]. Wang et al. 's study found that FBXO6 can reduce the expression of MMP14 protein through ubiquitination and subsequent proteasome degradation, thereby reducing the secretion of normally functioning MMP13, and thus reducing the degradation of chondrocytes ECM [49].

## 3 Ubiquitination regulates senescence, apoptosis and autophagy of osteoblasts and FLSs

Bone anabolism mediated by osteoblasts and bone catabolism mediated by osteoclasts are two factors that affect bone homeostasis [52,53]. Studies have shown that in the course of OA, the apoptosis level of osteoblasts increases, resulting in the imbalance of bone anabolism and bone catabolism, resulting in the loss of the original stress support ability of subchondral bone, and the formation of osteophytes in subchondral bone, resulting in the destruction of joint structure [[54], [55], [56]]. Consequently, subchondral osteoporosis emerges as a significant contributor to the pathogenesis of OA. This condition, characterized by the deterioration of subchondral bone density and structural integrity, exacerbates the progressive degeneration of articular cartilage, thereby intensifying the clinical manifestations of OA. TRIM33 is a member of the TRIM (tripartite motif) family, which acts as an E3 ubiquitin ligase with N-terminal zinc finger domains for potential ubiquitin ligase activity, one or two b-BOX motifs, and a curly helix region [57]. Studies have shown that the expression of osteoblasts in patients with osteoporosis is significantly down-regulated and positively correlated with the bone mineral density of patients [58]. Silencing the TRIM33 gene leads to the degradation of FOXO3a in normal osteoblasts [59]. The latter belong to the FOXO (Forkhead box proteins) transcription factor family, which are mainly involved in cell growth, apoptosis, differentiation and autophagy, and are highly expressed in osteoblasts [59,60]. Mice specifically deprived of FOXO3a showed increased apoptosis of osteoblasts, whereas overexpression of FOXO3a inhibited apoptosis of osteoblasts and increased the ability of osteoblasts to resist oxidative stress [60]. TRIM33-FOXO3a ubiquitination degradation signaling is a pathway that relies on the cell itself to clear excess oxygen free radicals, thereby resisting cell apoptosis [59]. This resistance and defense mechanism to oxidative stress is essential for maintaining bone mass.

FLSs are the main cell type in the joint lumen, secreting lubricants and metabolites that maintain joint function. In osteoarthritis, overgrowth and abnormal apoptosis of FLSs lead to joint inflammation and cartilage degeneration [61]. In OA patients, the FLSs will become over-aged and even apoptotic, resulting in a lack of lubrication at the joint, which leads to pain and wear of the articular cartilage, further aggravating inflammation [62]. Ubiquitination is involved in the regulation of inflammatory response by regulating the expression of inflammation-related genes, such as NF-κB and COX-2, in FLSs [63]. At the same time, the regulation of FLS apoptosis is also affected by the ubiquitination pathway. For example, the ubiquitination status of Bcl-2 family proteins determines whether a cell enters the apoptosis program [64]. BAX protein (Bcl-2 associated X protein) and BCL-2 protein (B-cell 2 protein) belong to the Bcl-2 protein family, the homeostasis between BAX and BCL-2 is a critical determinant of chondrocyte survival and apoptosis in OA [65]. The former is a pro-apoptotic protein, which mainly mediates apoptosis, while the latter is an anti-apoptotic protein, which resists apoptosis [65]. OA creates a microenvironment of inflammation and oxidative stress in the patient's joint cavity, which may be the main reason for the increased expression of BAX protein [65]. Studies have shown that the expression of BAX protein is upregulated in articular chondrocytes of OA rats, which leads to the failure of the BCL-2 protein to perform its normal function, leading to excessive apoptosis of FLSs [66]. Similarly, aging and apoptosis of FLSs can promote the MAPK pathway, leading to the degradation of chondrocyte ECM, and then aggravating inflammation, forming a vicious negative feedback cycle [67]. DUSP1 (dual-specificity phosphatase-1) is reduced in articular chondrocytes of OA patients, and in fact, DUSP1, through its phosphatase activity, regulates the activity of kinases such as MAPK and ERK, affecting cell growth, differentiation, and apoptosis [68]. In OA, the decreased expression of DUSP1 may lead to the abnormal activation of key kinases in certain signaling pathway nodes, thus promoting the inflammatory response of FLSs and the degradation of ECM [68]. PENG et al. also demonstrated that overexpression of DUSP1 in OA FLSs inhibited the activation of MAPK and JNK pathways and the expression of MMP13 and COX-2, SKP2 can regulate the ubiquitination of DUSP1 and subsequent proteasome degradation [69]. Existing studies have shown that MiR-337-3p can inhibit the ubiquitination of DUSP1 by binding to SKP2, thereby up-regulating the proliferation of FLSs and protecting them from inflammatory damage [70]. Consequently, the modulation of the ubiquitin levels associated with apoptotic factors presents a promising avenue for the development of therapeutic strategies aimed at addressing OA. This approach is grounded in the premise that altering the balance between these key apoptotic regulators could influence the pathophysiological processes underlying OA, potentially leading to a reduction in joint degeneration and an improvement in overall joint health.

## 4 Ubiquitination regulates macrophage polarization

Frequent and excessive mechanical stress can cause wear of articular cartilage, and the main components of articular cartilage extracellular matrix are type II collagen and proteoglycan [71]. The inflammatory response associated with OA is primarily due to the innate immune response induced by synovial macrophages in the joint, which are specifically activated into a classically activated proinflammatory phenotype (CD80, CD86, and CD11b as M1 surface markers). In contrast, selectively activated anti-inflammatory phenotypes (CD163 and CD206 as M2 surface markers) are responsible for clearing inflammation [72].

The fragments of type II collagen, generated through wear, function as Damage-Associated Molecular Patterns (DAMPs) within the synovial cavity, and are recognized by the pattern recognition receptors on macrophages [73]. This interaction prompts the macrophages to undergo M1 polarization, following which they release an abundance of inflammatory mediators, including but not limited to IL-1 and TNF-α [74]. This cascade is deemed to be a pivotal mechanism initiating the inflammatory response that characterizes the onset of OA pathology. There are a large number of M1-type macrophages in the synovium of OA patients, and the inflammatory factors secreted by them include TNF-α and IL-1β, which can trigger the aging and apoptosis of chondrocytes and FLSs by activating the inflammatory signaling pathway in them [75]. In addition, such changes also induce cell autosecretion of IL-1β and TNF-α, resulting in the production and secretion of NO, PGE2 and MMPs, which inhibit the chondrocyte ECM [76].

The role of TNF-α in the pathogenesis and progression of OA and the regulation of macrophage polarization is relatively complicated. On the one hand, TNF-α, as a TH1 cytokine, can induce classical activation of macrophages, which leads to inflammation [77]. On the other hand, by binding to the TNF-R1 receptor on the cell membrane, it recruits the adaptor proteins TRADD and FADD, initiates the NF-κB signaling pathway, and promotes the expression of apoptosis-related proteins and inflammation-related proteins [78]. This not only intensifies the apoptosis of chondrocytes and synovial cells, but also further promotes the classical activation of macrophages, forming malignant negative feedback regulation. When TNF-α binds to TNF-R1, it induces TNF-R1 transmembrane domain recruitment of the junction proteins TRADD (TNF receptor-associated death domain), TRAF2/5 (TNF receptor-associated factor 2/5), cIAP1/2 (apoptotic protein inhibitor 1/2), and RIP1 (receptor-interacting serine/threonine-protein kinase 1), formation of initial complex 1. Therefore, in order for TNF-α to play its ligand-related role, it needs to bind to TNF-R1 to trigger the combination of complex 1, and after the combination of complex 1, RIP1 can be successfully ubiquitination and degradation, so as to continue to transmit signals downstream [79].

RNF8 (Ring Finger protein 8) is a class of E3 ubiquitin ligases with a forkhead-associated (FHA) domain at the N-terminal. The C-terminal has a Really Interesting New Gene (RING) domain common in ubiquitin ligases [80]. RNF8 catalyzes the specific assembly of K63/K48-linked polyubiquitin chains on substrate proteins, thereby contributing to their nucleic acid translocation or subsequent proteasomal degradation [81]. Jurgen Fritsch et al. found that RNF8 can ubiquitinate to degrade TNF-R1, which occurs after TNF-α ligand binding and before TNF-R1 internalization, and is necessary for TNF-α-related signaling to be transmitted downstream. The absence of RNF8 in cells will significantly reduce the formation of complex 1, thereby reducing TNF-α signal-induced activation of caspase-8, caspase-3/7, A-SMase and subsequent apoptosis [82]. Therefore, RNF8 may be a novel therapeutic target to block TGF-α signaling, thereby reducing inflammation and inhibiting apoptosis of chondrocytes and synovial cells. CARP-1/-2 (Caspase-8-and caspase-10-associated RING Protein-1 or 2) is a class of E3 ligases containing RING domains. Ubiquidize apical caspases and target them for subsequent proteasome degradation [83]. CARP-1/-2 mediates the ubiquitination degradation of RIK1, which is critical in TNF-αsignaling, and with the K63 ubiquitination of RIK1 and subsequent proteasome degradation, TRADD in complex 1 exposes the death domain, Further, FADD(Fas related death domain), caspase-8 and caspase-10 are recruited to transmit apoptosis signals [84]. Liao et al. also confirmed that knocking down CARP-1/-2 stabilizes the level of RIP1 protein, but also increases the activation of NF-κB signaling [85]. Therefore, the results of regulation of RIK1 by CARP-1/-2 ubiquitination are diverse, and the relevant mechanisms should be comprehensively considered and explored when developing drugs targeting this pathway.

IL-1β can stimulate the production of IL-6 by macrophages, and IL-6-STAT3 signaling pathway is the main signaling pathway that promotes the classical activation of macrophages [86]. SYVN1 is an E3 ubiquitin ligase that plays an important role in the recognition and elimination of misfolded and non-functional proteins produced by the endoplasmic reticulum [87]. It maintains cellular immune homeostasis, inhibits apoptosis, and reduces chronic inflammation by recognizing specific protein substrates and using subsequent proteasomes to inhibit the unfolded protein response. One of the ways to reduce chronic inflammation is that SYVN1 uses its own E3 ubiquitin ligase activity to down-regulate the expression of STAT3, thereby reducing the activation of IL-6-STAT3 signaling pathway [88]. In addition to inhibiting IL-1β, it can also regulate the polarization of macrophages by inhibiting chondrocytes or synovial cells from secreting IL-1β. Notably, the aforementioned FBXO3 can promote the expression of IL-1β in cells through relevant ubiquitination regulation. Deletion of FBXO3 or addition of antagonists such as MiR-219a-5p can significantly inhibit the action of FBXO3 and reduce the content of IL-1β and IL-18 [89].

In addition to ubiquitination, some deubiquitination processes can also affect the expression of IL-1β. USPS, or deubiquitination enzymes, can remove ubiquitin labels from ubiquitin-linked protein substrates and protect the substrates from ubiquitination degradation, thereby regulating certain cellular processes [90]. Downregulation of USP3 expression in OA led to overactivation of the NF-κB pathway and subsequent overexpression of IL-1β. The key protein involved, TRAF6, is a key adaptor of the NF-κB pathway, and its ubiquitination degradation is a prerequisite for the subsequent release of NF-κB, but this ubiquitination degradation pathway can be canceled by overexpression of USP3 in OA [91]. USP5 can also stabilize TRAF6 by deubiquitination, but contrary to the results of USP3, overexpression of USP5 increases the activation of NF-κB pathway, promotes the release of NF-κB, and then up-regulates the secretion of pro-inflammatory cytokines such as IL-1β in rheumatoid arthritis (RA) [92]. The contractional effects between USP3 and USP5 maybe because of the different mechanisms between OA and RA.

All the above-mentioned ubiquitination pathways can regulate the polarization of macrophages, indicating that the inflammatory microenvironment shaped by OA in the joint cavity has relatively complex regulation on macrophages, FLSs and chondrocytes, and targeting a single ubiquitination or deubiquitination target may not have a good therapeutic effect on OA in clinic. Further research is warranted to elucidate the precise mechanisms by which such interventions could be implemented and to assess their efficacy and safety in preclinical and clinical settings.

## 5 Skeletal muscle and adipose tissue regulate OA through complex ubiquitination mechanisms

One of the reasons why OA is extremely difficult to cure is that its pathogenesis is very complex, and the complex factors affecting the OA process are not simply limited to the knee joint and joint-related diseases such as arthrofibrosis [93], but also other organs in the body, especially adipose tissue [94] and skeletal muscle [95].

The skeletal muscle is the largest organ in the human body to help the human body complete many complex mechanical activities through complex myotome combinations [95]. With the development of research, skeletal muscle is also considered to be a secretory organ, which also secretes a variety of cytokines, namely myokine, of which irisin is one of them [95]. Moderate to high-intensity exercise can significantly up-regulate the level of serum irisin, which regulates bone and cartilage metabolism through a complex mechanism. Irisin not only activates osteogenic differentiation through the activation of Wnt/β-catenin and AMPK pathways but also enhances osteoblastic transcription regulators such as osterix and RUNX2 [96]. Demonstration has been presented as consistently lower BMD in irisin-deficient mice [97]. Proper bone synthesis/metabolism is the primary prerequisite for maintaining normal joint function. For instance, voluntary exercise has been shown to increase the production of irisin in bone, and elevated circulating levels of irisin are associated with enhanced osteogenesis in mice [98]. On the contrary, excessive bone loss will lead to stress redistribution, which will lead to osteophytic hyperplasia and articular cartilage damage [54]. Since cartilage is the bearer of friction stress and shear stress, excessive mechanical stress can inhibit the expression of FBXW7 (F-box and WD repeat domain-containing 7) in chondrocytes and enhance OA [99]. The potential mechanism may be due to FBXW7 upregulating the expression of SOX9, COL2A1 and ACAN through suppressing the HIF-1a/VEGF pathway, thereby protecting articular chondrocytes [100]. In addition to regulating bone synthesis/metabolism, irisin also alleviates the inflammation of osteoarthritic chondrocytes by inhibiting the p38, Akt, JNK and NFκB signaling pathways [101]. In addition to the role of JNK phosphorylation in the progression of OA described earlier, JNK phosphorylation also phosphorylates other components of the AP-1 complex [102]. This complex mainly regulates the degradation of ECM of articular cartilage [103], and the related mechanism is mainly through JUNB binding to FBXO21, thus activating ubiquitination regulation [104]. FBXO21 is a subunit of ubiquitin E3 ligases that facilitates the degradation of P-glycoprotein and EID1 through ubiquitination, and it activates the JNK and p38 signaling pathways [104]. In chondrocytes, the expression of FBXO21 is mainly regulated by JUNB, which can directly bind to the promoter of FRXO21, and play the role of enhancing apoptosis and catabolism by inhibiting autophagy, thus aggravating OA [104]. Therefore, skeletal muscle not only directly affects cartilage and bone by coordinating mechanical stress, but also constructs a complex regulatory network by secreting cytokines such as irisin that regulate OA.

Obesity is another important factor contributing to OA. On the one hand, obesity will lead to weight gain, which will aggravate the mechanical wear of the knee joint. On the other hand, the highly expressed white adipose tissue in obesity will secrete many adipokines such as visfatin that participate in the progression of OA [94]. Visfatin is a protein derived from fat cells, and the serum visfatin content of obese individuals is higher than that of normal people, and this change can be reversed by weight loss [105]. Moreover, visfatin induces the production of pro-inflammatory factors such as IL-1 and TNF-α, thus mediating many of the inflammations associated with adipocytes [106]. During the development of OA, visfatin, on the one hand, inactivates the glycogen synthase kinase 3β (GSK3β) by activating the p38 signaling pathway, destroying the network structure formed by microfilament microtubules in chondrocytes, resulting in loss of cell elasticity and adhesion, and on the other hand, up-regulates the level of phosphorylated ERK protein [107]. Overexpression of this protein will inhibit the autophagy repair of chondrocytes. If the OA chondrocytes are treated with ERK protein inhibitor U0126, the damaged autophagy of chondrocytes can be restored [108]. In addition, phosphorylated ERK protein is also closely related to JUNB-FBXO21 axis, which is associated with autophagy and ubiquitination as previously described [104]. Apelin is also an adipokine, which can be detected in the synovial fluid of OA patients as an indicator of the degree of OA development [109]. Its role is mainly through its specific ligand APJ, upregulation of MMP-1/-3/-9 expression and IL-1β proinflammatory cytokine expression [109]. Most critically, apelin also promotes the expression of VEGF in synovial fibroblasts, leading to the generation of synovitis and forming malignant feedback in the progression of OA [110]. However, apelin has a complex inhibitory effect on miR-150-5p [110]. Studies on non-small cell lung cancer have shown that miR-150-5p can prevent the autophagy of cancer cells by blocking the fusion of autophagosomes and lysosomes [111]. Similarly, high levels of miR-150-5p inhibit autophagy by suppressing the interaction between SIRT1 and p53 in a mouse diabetic model [112]. Therefore, the inhibitory effect of apelin on miR-150-5p may have a positive effect on the regulation of autophagy. APN (Adiponectin) is also an adipokine secreted by adipocytes, which has insulin-sensitizing, anti-atherosclerosis, anti-inflammatory, and anti-angiogenic effects [113]. Studies have shown that APN peptide regulates the AMPK/GSK-3β pathway to relieve oxidative stress and inhibit the activation of NLRP3 inflammasome [114]. It also inhibited apoptosis by regulating the expression of anti-apoptotic protein Bcl-2 in mouse adipose tissue [115]. Inhibition of ERK1/2 pathway activation was found in the constructed mouse model of animal obesity, which led to downregulation of plasma APN content, but the expression of APN gene level was not affected, suggesting that ERK1/2 pathway may mediate the degradation of APN protein level, which is indeed the case by applying MG132. A potent protease inhibitor prevents APN degradation even when activation of the ERK1/2 pathway is inhibited [116]. Leptin is the main adipokine secreted by adipocytes, which regulates reproduction, glucose metabolism, hematopoietic and interacts with the immune system in the human body [117]. Its function is mainly through binding with leptin receptor Ob-Rb to activate downstream signaling pathways. Studies have shown that the leptin receptor Ob-Rb is highly expressed in OA cartilage, thus over-activating the leptin pathway leads to cartilage degradation. If the cartilage cells overexpressing leptin receptor Ob-Rb are treated with normal physiological dose of leptin *in vitro*, the P53/P21 pathway will be activated to promote chondrocyte senescence [118]. The expression of leptin receptor Ob-Rb is regulated by USP8. Overexpression of USP8 can significantly up-regulate the expression of the leptin receptor Ob-Rb on the cell surface, and acute stimulation of cultured neurons with leptin can also up-regulate the expression of USP8 [119]. This suggests that USP8-mediated deubiquitination is necessary for leptin pathway activation. However, we must emphasize that the function of leptin in the human body is diverse and complex, so whether the deubiquitination site can be applied to the clinic needs more thorough research.

In general, the cause of OA is extremely complex, and various factors will form a complex regulatory network to interfere with the normal autophagy of cells and interfere with the regulation of ubiquitination. In addition, local inflammation such as synovitis and adipocyte-mediated inflammation will lead to the increase of the content of inflammatory factors near the joint, and these inflammatory factors will lead to the expansion of the inflammatory range after being absorbed by healthy cells, and interfere with the ubiquitination of normal proteins and autophagy, thus forming malignant feedback.

## 6 Ubiquitination of related drugs is used to treat OA

This section delves into the emerging pharmacological landscape to unveil the potential application of ubiquitination in OA treatment. It highlights a suite of compounds—including Digoxin, 6-Gingerol, Resveratrol, Spermidine, Alpinetin and Melatonin—that have been recently introduced or are under investigation for their potential to mitigate OA pathology through the regulation of protein ubiquitination (Table 1). These agents, each with unique mechanisms of action, offer promising avenues for the development of targeted, disease-modifying OA treatments.

**Table 1:** Cells and signals affected by Digoxin, 6-Gingerol, Resveratrol, Spermidine, Alpinetin and Melatonin.

Drug name	Mechanism of Action	Ubiquitin/Deubiquitinating enzymes	Target molecule/signal pathway	Kind of cells	Reference
Digoxin	Inhibits synovial macrophage M1-like polarization	Usp3 and Sox5	miR-146b-5p/Usp3&Sox5 axis	synovial macrophage	[120]
6-Gingerol	Upregulates the protein level of Usp49, which in turn directly inhibits the Wnt/β-catenin signaling cascade	Usp49	Wnt/β-catenin signaling	chondrocytes	[121]
Resveratrol	Inhibits IL-1β-induced IkBa degradation in chondrocytes	IκB	NF-κB	chondrocytes	[122]
Spermidine	Prevents TNF-α-induced NF-κB/p65 activation by suppressing RIP1 ubiquitination	RIP1	NF-κB/P65	synovial macrophages	[123]
Alpinetin	Anti-inflammatory effects in chondrocytes by interfering with the NF-κB/ERK1/2 signal pathway	IκB	NF-κB/ERK	chondrocytes	[124]
Melatonin	Upregulates the expression of miR140, a downstream target gene WWP2 that encodes the E3 ubiquitin ligase	WWP2	MicroRNA-140	chondrocytes	[125,126]

Digoxin has been approved by the FDA for the treatment of heart failure, atrial fibrillation, supraventricular tachycardia, and cardiomyopathy [127]. With the in-depth study, it was found that Digoxin can also promote the anabolism of OA chondrocytes through LRP4 (low density lipoprotein receptor associated protein 4), which makes this drug approved for clinical use in the treatment of OA [128]. Jia et al. found that Digoxin can inhibit the classical activation of synovial macrophages in OA patients, and also affect the exosome components of macrophages and macrophages after classical activation. The inflammatory microenvironment shaped by OA can be reduced through the miR-146b-5p/Usp3-Sox5 axis [120].

Ginger has long been known to have a wide range of medicinal properties, and is often used to treat conditions including nausea, asthma, arthritis, gastrointestinal disorders, and headaches. The compounds that embody the medicinal use of ginger are mainly the large amount of gingerol and gingerol contained in it [129]. 6-gingerol inhibits inflammation by upregulating the expression of MKP5, while reducing the activation of the NF-κB pathway, and alleviates oxidative stress by providing electrons and scavenging oxygen free radicals [130]. In addition, the study of Yang et al. found that 6-gingerol can significantly up-regulate the level of USP49 in OA chondrocytes, and the expression of the latter is significantly reduced in OA patients, resulting in the failure of Axin protein deubiquitination and excessive accumulation leading to apoptosis of chondrocytes [121].

Resveratrol is a stilbene molecule extracted from natural plants, which has been widely used in the food and pharmaceutical industries because of its many beneficial properties, such as antioxidant, anti-inflammatory, cardiovascular protection, anti-cancer, neuroprotection, anti-aging, etc [131]. Shakibaei et al. found that Resveratrol could inhibit the expression of MMP-3 and COX-2 in human OA chondrocytes induced by IL-1β. The main reason for this phenomenon is that Resveratrol inhibits the ubiquitination degradation of I-κB and the related proteasome function induced by IL-1β [122].

Spermidine has a wide range of anti-inflammatory, anti-aging, and anti-cancer properties due to its physiological induction of autophagy by inducing protein deacetylation and triggering physiological autophagy [132]. Currently, Spermidine is considered a modulator of ubiquitination. Chen et al. found that Spermidine could inhibit the ubiquitination degradation of RIP1, thus blocking TNF-αsignaling [123]. Recent studies have also found that Spermidine can induce macrophages to selectively activate into anti-inflammatory phenotype, and even transform classically activated pro-inflammatory phenotype macrophages into selectively activated anti-inflammatory phenotype macrophages, and can also reduce synovial cell senescence and synovial inflammation [133].

Alpinetin is a kind of natural flavonoid, which is contained in many herbs. Alpinetin has anti-tumor, anti-inflammatory, liver protection, cardiovascular protection, lung protection, antibacterial, antiviral, neuroprotective pharmacological effects [134]. Gao et al. showed that Alpinetin can effectively inhibit the phosphorylation of I-κB, thereby inhibiting its subsequent proteasome degradation, and to a certain extent, blocking the nuclear translocation of NF-κB, thereby reducing the production of MMP13 and ADAMTS5, and protecting chondrocytes from inflammation [124]. In addition to its protective effect on chondrocytes, Alpinetin can also improve bone loss through the P38/PI3K signaling pathway, which is beneficial for balancing stress stimulation between bone and cartilage and reducing osteopathic hyperplasia [135]. At the same time, Alpinetin can also inhibit the expression of PI3K protein. The PI3K/AKT/FOXO1 signaling pathway is a common apoptosis regulation pathway in cells, and the expression of PI3K protein and phosphorylation of AKT can up-regulate the apoptosis of chondrocytes [136]. As a transcription factor, FOXO1 not only regulates apoptosis but also participates in a variety of biological processes including autophagy, antioxidant stress, cell cycle, metabolism and immunity depending on the upstream stimulus and downstream target [137]. Although Alpinetin can be used clinically to treat OA, there may be more detailed mechanisms worth exploring.

As one of the hormones secreted by the pineal gland of the brain, Melatonin has a wide range of anti-inflammatory, antioxidant, and anti-aging properties [138,139]. In the development of OA, Melatonin can up-regulate the expression of COL2A1 and aggrecan by inhibiting the expression of MMPs and ADAMTS [138]. Moreover, Melatonin prevents TGF-β1-induced cellular processes by inhibiting both Smad and non-Smad signaling pathways through the suppression of ROS-mediated mechanisms [140]. Similarly, Melatonin inhibits the phosphorylation of IκB, leading to the blocking of the NF-κB signaling pathway, and its regulation of NRF2 protein levels synergistically inhibits the progression of inflammation [141,142]. More importantly, Melatonin also up-regulates the expression of miR140 [125], a downstream target gene WWP2 that encodes the E3 ubiquitin ligase [126]. In the OA murine model, WWP2 protein levels were significantly down-regulated, and mice lacking the WWP2 gene developed spontaneous osteoarthritis. The substrate of WWP2 is RUNX2, and inhibition of its protein content can reduce the expression of the ADAMTS5 gene, thus reducing the degradation of cartilage ECM [143].

## 7 Conclusion and perspective

In conclusion, ubiquitination and deubiquitination play an important role in the pathogenesis and progression of OA by controlling the abundance of key proteins or regulating signaling pathways. The high intensity mechanical stress causes the wear of the chondrocyte ECM, the M1 polarization of macrophages leads to the initial inflammation in the joint cavity, and the progressive inflammation and oxidative stress in the microenvironment lead to the aging and apoptosis of chondrocytes, FLSs and osteoblasts, making the joint unable to perform normal functions. However, the above factors are only part of the reasons why inflammation continues to progress and cartilage defects in joints are difficult to repair. Many other factors have also been confirmed to participate in the pathogenesis and progression of OA, and are also regulated by ubiquitination and deubiquitination.

Obesity has been proven to be one of the most important factors in the occurrence and development of OA. In addition to the mechanical stress caused by joint overload caused by weight rise, the hormone disorder brought by obesity, insulin resistance, and the incoordination of innate immune response and adaptive immune response caused by obesity will also promote the occurrence and development of OA [144]. This inevitably provides us with a new target for the treatment of OA, but the detailed pathway of ubiquitination or deubiquitination is still to be explored.

Physical inactivity and reduced exercise following the presence of mild OA have been shown to promote the occurrence and development of OA [145]. In the process of exercise, except skeletal muscle secretes a variety of muscle factors and adipose tissue secretes lipid factors, heart tissue secretes many cardiovascular factors that may also be important. Irisin is not only found in the skeletal system but also in cardiovascular tissues. For example, irisin can reduce myocardial ischemia/reperfusion injury, endoplasmic reticulum stress, and ROS production and maintain mitochondrial homeostasis by upregulating mitochondrial ubiquitin ligase (MITOL) [146]. The important influence of mitochondrial dysfunction on the progression of OA has been described above. Many studies have shown that irisin plays an important role in maintaining mitochondrial homeostasis [147,148]. Therefore, it may be a highly effective target for the treatment of OA by alleviating oxidative stress and modulating mitochondrial homeostasis. Lactic acid is the product of the anaerobic respiration of cells during physical activity, and its content represents whether the cell is properly using glucose and glycolysis pathways, which inevitably involve hormone homeostasis such as insulin, glucagon, adrenaline, etc. [149]. These hormones have certain regulatory effects on chondrocytes, including multiple ubiquitination and deubiquitination pathways. However, lactic acid alone also has a complex influence on the occurrence and development of OA. *In vitro* treatment of OA chondrocytes with 100 mM lactate can promote the expression of COL2A1, but the down-regulation of microenvironment pH caused by lactic acid has certain adverse effects on chondrocyte proliferation and ECM expression [150]. Therefore, the use of lactate or lactic acid as a target for the treatment of OA still needs more research and consideration.

**Scheme 1:**
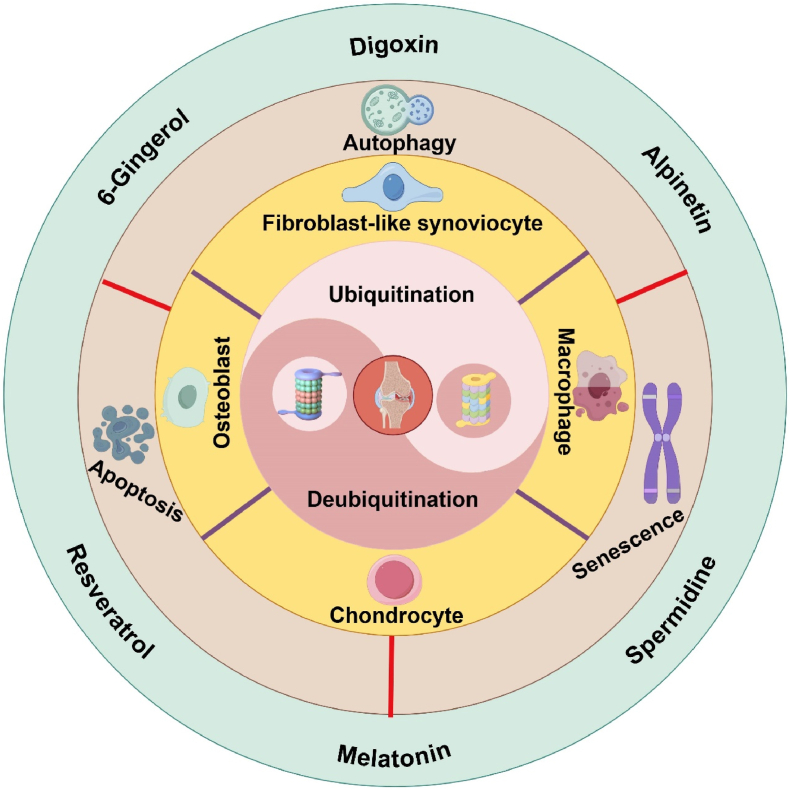
Ubiquitination and deubiquitination participate in the homeostasis between healthy joints and osteoarthritic joints through regulating senescence, apoptosis and autophagy of chondrocytes, fibroblast-like synoviocytes, osteoblasts, macrophages, skeletal muscle and adipose tissue. Digoxin, 6-Gingerol, Resveratrol, Spermidine, Alpinetin and Melatonin are potential targeted drugs. By Figdraw.

In short, the occurrence and development of OA are undoubtedly complex, and these factors converge into a complex and huge regulatory network in the body. We cannot determine which ubiquitination or deubiquitination regulatory mechanism has the greatest impact on OA, but we will continue to explore and find relevant ubiquitination and deubiquitination targets. In particular, targeting the ubiquitination pathway in the processes of senescence, apoptosis, and autophagy in chondrocytes, FLSs, osteoblasts, as well as the polarization of macrophages, is essential for OA treatment. Potential targeted drugs include Digoxin, 6-Gingerol, Resveratrol, Spermidine, Alpinetin, and Melatonin (Scheme 1). Intriguingly, modulating the contents for these ubiquitination-related targets in the serum through exercise will provide a new idea for OA treatment or prevent OA from deteriorating continuously.

## Declaration of competing interest

The authors declare that they have no known competing financial interests or personal relationships that could have appeared to influence the work reported in this paper.
